# Notes and News

**Published:** 1886-10-23

**Authors:** 


### NOTES AND NEWS.

COLLECTED AND COMMUNICATED.

MR. E. W. WOODHOUSE, M.B., C.M., has been appointed resident house surgeon to the District Hospital, West Bromwich.

MESSRS. WORTHINGTON AND ELGOOD, Manchester, have been appointed architects to the new hospital at Harrogate, the contractors for which are Messrs. Ives and Co., Shipley.

MISS INGALL, formerly of the Miller Hospital, Greenwich, has been appointed Matron of the London Fever Hospital, in the room of Miss Mapleton, resigned.

THE grand entertainment in aid of the funds of the Metropolitan Free Hospital, at the opening of the North London Colosseum, has been unavoidably postponed from the 16th to the 30th inst.

MISS MINNIE CARLE MAGDSHON has won the entrance scholarship at the London School of Medicine for Women, the value of which is £30. No less than twenty-two new students have entered for the full professional curriculum, and five for classes for private study. Of the twenty-two professional students, ten have passed the preliminary scientific M.B. examination of the London University, and three the preliminary Arts examination of the Royal University of Ireland.

THE Kent County Ophthalmic Hospital, at Maidstone, has just been the fortunate recipient of £10,000, through the death of Mrs. R. Mount, widow of the late Mr. Richard Mount, of Canterbury. A sum of £30,000, it is said, passes under the same will to a similar institution in London.

OWING to the appointment of the Marquis of Londonderry to the post of Lord Lieutenant of Ireland, the Earl of Durham has undertaken to open the new buildings recently erected in connection with the Durham County Hospital, at a date not yet fixed. The architect is Mr. C. Hodgson Fowler, M.A., F.S.A., of Durham. About £6,000 has been spent upon the new buildings.

THE District Hospital, West Bromwich, of which Mr. W. H. Lehmann is the secretary, contains fifty beds, thirty-one of which were occupied on an average throughout the year ended June 1886. It is one of the most actively worked of recent provincial hospitals. The number of in-patients admitted during last year was 497, whilst the out-patients amount to 5,652, of whom 1,976 were cases of accident or emergency.

THE Friendly Societies of Stratford have raised £500 by means of concerts and demonstrations, for the building fund of the proposed hospital for West Ham. The financial account shows that the recent bazaar realised £1,063, so that the whole of the £3,000 required to erect the new hospital is now forthcoming, to the infinite credit of all concerned.

ABINGDON is justly proud of Mr. J. C. Clarke, the noble donor of the new Cottage Hospital, which is now nearing completion. Up to the present time, Abingdon and the neighbourhood has been very deficient in hospital accommodation, and the new institution will, we have no doubt, prove a vast boon to the poor. Cottage hospitals, it is true, are not without their drawbacks, but they have nevertheless already done much to alleviate suffering, and their tried usefulness will be readily acknowledged.

WE have not referred in THE HOSPITAL to "Sister Dora," for we have felt that the sum of praise which has been echoed by provincial and metropolitan journals will keep her memory green for many years to come. If we are not quite prepared to endorse the expression of the Mayor of Walsall, that "Sister Dora was one of the grandest and most illustrious women that ever lived," we fully recognise the worth of her devoted and disinterested labour on behalf of the sick and the poor. Miss Dorothy Wyndlow Pattison has died "all too soon," but we have in our midst at this moment several other Sister Doras, who will hand down the lamp of Christian philanthropy to burn perhaps yet brighter. The Dora celebration will, we think, be unsatisfactory, unless it is made to bear fruit in the direction of the foundation of an order of merit for those women who labour in the hospital field, unless they can be included in an existing order in this year of Jubilee.

---

Fresh fruit is one of the most acceptable gifts which can be made to a hospital, yet our great charities seem to come off very badly in this respect. How glad the superintendents and matrons would be of a few fruit-offerings just now, or, indeed, at any time! Much of the neglect from which many of our hospitals suffer arises rather from the ignorance which prevails of their actual wants, than from a stolid indifference on the part of the people. One of the missions of The Hospital will be to make known the needs of our institutions, for with knowledge only will come a wider and more practical support.

---

The cry of the hospitals is, 'tis true, "Give, give," but the givers are few and the recipients many. At a time when fruit is so plentiful in many places that we are informed in some districts it does not pay for the gathering, many people who can give but little, perhaps, in money, might send something of the bounteous produce of the soil—salad, fruit, and so forth. Again, how notorious is it that country folk store up their old linen in quantities destined never to be used! A few bundles of old linen would be most acceptable at the hospitals. If men or women are anything, let them be practical. Let them strive to turn everything to a useful purpose for the benefit of their fellows. The burden of our hospitals should fall on the houlders [illegible] and not, [illegible] now, on those [illegible] the comparatively few.

---

While we are talking about practical people, another thought occurs to us. In passing through the wards of a well-known hospital the other day, the walls were seen to be hung with pictures,—but such pictures! Common oleographs of dead fish, and the like, are not the subjects most calculated to elevate the mind. Yet the decoration of a hospital ward should be a matter of no small moment. A picture or a text, which day by day hangs before the bed of a poor sufferer, will force itself upon his observation, and if it has a lesson to teach, it will probably teach it. The picture should therefore have its mission, which may chance hereafter to have a biding effect upon the patient. The hospitals can do much to elevate the masses, and to this end the donors of pictures and books should give those which tend to better things.

---

"Nurse W." sends us the following as an item of News (*sic*) for the Prize Competition :—

"Mental Excitement.—Bad news weakens the action of the heart, oppresses the lungs, destroys the appetite, stops digestion, and partially suspends all the functions of the system. An emotion of shame flushes the face; fear blanches it; joy illumines it; and an instant thrill electrifies a million of nerves. Surprise spurs the pulse into a gallop. Delirium infuses great energy. Volition commands, and hundreds of muscles spring to execute. Powerful emotion often kills the body at a stroke. Chilo Diagoras and Sophocles died of joy at the Grecian games. The news of a defeat killed Philip the Fifth. Eminent public speakers have often died in the midst of an impassioned burst of eloquence, or when the deep emotion that produced it suddenly subsided. Largrave, the young Parisian, died when he heard that the musical prize for which he had competed was adjudged to another."

We hope "Nurse W." will not share young Largrave's fate, when she hears the prize has been adjudicated to another!

---

Wednesday week was a great day for the town of Burnley and its inhabitants. It is rare that this part of Lancashire is favoured with a sight of Royalty. The district in which Burnley is situated has no peculiar attractions save for those interested in cotton. Besides, it is off the main railway line, which possibly accounts to some extent for the people being unaccustomed to the presence of the great ones of the earth. But the patronage of Royalty is well-timed, as recognising the really creditable manner in which the Burnley people have responded to the call of charity. Burnley and its immediate neighbourhood comprise a population of well-nigh 120,000 people, for whom no suitable hospital existed until Wednesday's ceremony. All classes, however, responded so generously to the appeal that was made three years ago, that it is expected the hospital will probably be opened, not only free of debt, but with a goodly balance in hand towards the maintenance expenses. We are informed that over £20,000 was expected to be subscribed before the opening ceremony, and of this sum the working people have contributed about £5,000. Here is indeed an example of a much-needed work being well and speedily done.

There are other reasons which make the opening of the Burnley Hospital and its future history matters of general interest. It is built upon what is known as the circular system, the other notable examples of this plan of construction being two military hospitals at Gravesend and Liverpool respectively, the Antwerp Civil Hospital, the Miller Hospital, Greenwich, and the new wards at the Hampstead Infirmary. The same system has been introduced into America at the John Hopkins Hospital, Baltimore, where some of the wards are octagonal, and at the Cancer Hospital, New York. Dr. J. S. Billings, of Washington, the greatest American authority, prefers an octagonal to a circular ward; but this is, after all, a detail, as both shapes are intended to obviate the difficulty of warming and ventilating all parts of a hospital ward equally, which is found insuperable in the old-fashioned parallelogram wards now so general. Experience can therefore alone decide between the octagonal and the circular ward.

---

Who shall say that the popularity of hospitals is decreasing in the provinces after the demonstration at Burnley last week? In addition to special trains from Manchester, Todmorden, Rochdale, and other places, no fewer than fifty-four other special trains arrived at Bank Top Station in the course of the day. The Tramway Companies did an amount of business wholly unprecedented in the two days during which the hospital festivities were continued. 17,580 passengers were carried by the tramways, and the receipts amounted to upwards of £180—that is, to an increase of an ordinary day's receipts of over £140. The excursion trains brought at least 18,000 people into Burnley on the opening day; the proceedings throughout were enthusiastic, and the interest taken in them by all classes may be gathered from the fact that between ten and eleven thousand copies of a special edition of the local paper are said to have been sold. All this is very satisfactory, and cannot fail to prove gratifying to Prince Victor and his illustrious father.

---

The renewal of fresh air in every part of a ward is becoming every day of more importance, as our knowledge increases of the way by which diseases due to germs are produced. Stagnant corners are difficult to purify. In like manner the heating is always a difficult problem. It is impossible that all parts of a parallelogram-shaped ward can be heated to an equal degree. Patients lying on either side of a fire, for instance, are too warm, while those remote from the fireplace suffer from the cold. Professor John Marshall, F.R.S., was the first person in England to advocate the erection of wards built on the circular plan. Amongst many advantages, he drew attention to the facility with which an extraction-shaft could be constructed to act equally in drawing foul air out of every part of a ward, whilst a system can be devised by which all the inmates would be kept in an uniform temperature. The plans for the Burnley Hospital were thrown open to competition, and Captain Douglas Galton was appointed to select the best and most suitable. The successful plan was one designed with parallelogram-shaped wards, and an alternative plan on the circular system was left unnoticed by the assessor. The people of Burnley had, however, heard much of the advantages claimed for the circular system, and in the end it was decided to adopt it.

---

The fourth Annual Report has just been issued of the Sidcup Cottage Hospital, and is of a very encouraging character. Last year, fifty-eight in-patients were treated at this institution, which has now been established for four years. Colonel Beamish is the president of the hospital, Dr. Hanbury is the consulting surgeon, and the medical officers are Drs. Bull and Poole, and Mr. F. Shapley.

---

The Liverpool Corporation recently advertised for designs for a new Hospital for Infectious Diseases, to form part of a complete scheme for dealing not only with epidemics, but for ordinary outbreaks of disease. In answer to the invitation, some twenty-five designs were sent in. Messrs. Keith, Young, and Henry Hall, who have made hospital construction a study, were called in as professional assessors to assist the Hospital Committee, and they reported that in their judgment the design submitted by Messrs. Simpson and Allen, of London, was the best, and that of Col. Ellison, the second in order of merit. The first premium of £50 has accordingly been awarded to the first-named gentleman, and that of £25 to Col. Ellison.

---

The great advantages of cooking by gas have been strikingly exemplified at one of the London hospitals, where a complete set of apparatus by Messrs. Slater, of Holborn, has now been in use for some months. The actual measured saving in cost of fuel amounts to over 25 per cent., whilst the improvement in the character of the cooking, the saving of labour in the kitchen, and the reduction of the percentage of waste in the process of cooking, are all equally marked.

